# “The soul that goes to the doctors does not live:” disease and illness among nineteenth-century Russian peasants

**DOI:** 10.1590/S0104-59702024000100068

**Published:** 2024-12-16

**Authors:** Oksana Golovashina, Sergey K. Lyamin

**Affiliations:** i i Leading scientific researcher, Ural Federal University. Yekaterinburg – Russian Federation laomin@mail.ru

**Keywords:** Illness, History of medicine, Mentality of the peasantry, Annemarie Mol (1958, Enfermidade, História da medicina, Mentalidade campesina, Annemarie Mol (1958

## Abstract

The article deals with the representation of illness among Russian Orthodox peasants from the Russian Empire in the nineteenth century. Materials from ethnographic expeditions, folklore, nineteenth-century texts on treatments, memoirs, and publications in the local press are used as sources. Analysis of the sources allowed us to reach the following conclusions: the conception of illness among Russian peasants was constructed by various actors; rural doctors were the least influential among these actors; and illnesses were represented as a consequence of mixing the world of the living and the world of the dead or the action of anthropomorphic or zoomorphic entities, with treatment implying a return to the natural (“correct”) order of things.

## Resumo

O artigo trata da representação das enfermidades entre campesinos ortodoxos russos no Império Russo do século XIX. As fontes usadas compreenderam materiais de expedições etnográficas, materiais folclóricos, bem como textos do século XIX que falam sobre tratamentos, memórias e imprensa local. A análise das fontes nos levou às seguintes conclusões: o conceito de enfermidade entre os campesinos russos era construído por vários atores; médicos rurais eram os atores menos influentes; e as enfermidades eram representadas como uma consequência da mistura do mundo dos vivos com o dos mortos ou como a ação de entidades antropomórficas ou zoomórficas, com o tratamento implicando um retorno à ordem natural (“correta”) das coisas.

Practices in folk medicine, as considered by cultural anthropology and ethnography (Malinowski, 1926, 1948; Lévi-Strauss, 1963, 1978; Popov, 1903), have attracted the attention of researchers as a source for writing the social history of ethnic groups (Singer, Erickson, 2015; Williams, 2006; Candrea, 1999; Dmitrieva, 1999) and examining the influence of various factors on the development of medical knowledge (Sobo, Loustaunau, 1997; O’Connor, Hufford, 2002). While agreeing with the achievements of our colleagues, we propose to move away from a descriptive and instrumental approach to folk medicine and, instead, consider the very conditions and possibilities of representing illness in a situation dominated by the practices of folk medicine. We draw on the conclusions of Annemarie Mol, who argues that conceptions of illness are an assemblage of various actors (Mol, 2002). In contrast to researchers who evaluate traditional medical practices from the perspective of current scientific understandings of health and illness (Torri, Herrmann, 2010; Williams, 2006; Templing, 2017), we propose to look at them through the lens of direct participants and actors, intending not to reduce the effectiveness of traditional medicine to the placebo effect and psychological factors. We are interested not so much in the reasons for the success (or failure) of specific treatments as in the changing perceptions of disease and the conditions that contributed to these changes. Following Mol, we use the distinction between disease, as a physical bodily phenomenon, and illness, the categorization of which implies the consideration of various factors related to the life of the patient and his or her relatives, surrounding objects, and community.

The main sources for the study are previously published reports from ethnographic expeditions (Kochegarov, 1883; Makarenko, 1897; Popov, 1879) and nineteenth-century works on the treatments that existed among Russian Orthodox peasants (Krutovsky, 1911; Novoselsky, 1916; Molleson, 1869; Popov, 1903; Portugalov, 1868; Reformatsky, 1892). Additional sources include stories from local newspapers, published materials on folklore, and the memoirs and recollections of people who encountered folk medicine, in one way or another, in the nineteenth century.

We first analyze the role of various actors, the peculiarities of their interpretation of disease, and the practices of interaction with folk healers, priests, and doctors. We then describe what methods of treatment were offered to peasants in the nineteenth century and how the effectiveness of these treatments was determined. We must also note that our research relates only to Russian Orthodox peasants, since their everyday lives and conceptions are well represented in the ethnographic material and academic works from the period of interest to us; equally importantly, this ethnic and estate group constituted the majority of the Russian Empire’s population. Accordingly, when we write about peasants, we have in mind only Russian Orthodox peasants. We also understand that the Russian Empire of the nineteenth century was rather heterogenous in terms of its socio-economic composition; as such, our source base does not allow us to reach conclusions applicable to all Russian Orthodox peasants. As mentioned elsewhere (Vasil’ev, Vasil’ev, 2009, p.248-251), the standing of medicine, and consequently the means for treating illnesses, was different in Russia’s economically developed regions and those territories distant from the state’s center. While these differences were not fundamental, nineteenth-century anthropologists and ethnographers were more interested in the country’s northern and Siberian regions, since it was there that they could find preserved traditional conceptions of the world and medicine. Therefore, our source material mainly comes from these regions.

## Who treated illness?

The life of a Russian Orthodox peasant was supposedly a harmonious cycle based on the stages of agricultural labor. The peasant consciousness perceived any phenomenon that disturbed the cyclicity of life not just as a temporary difficulty, but as fatal disharmony, the result of someone’s evil intent or punishment for sin. Illnesses could, in many cases, lead to the loss of ability to work. Proverbs vividly demonstrate folk ideas about health as an important factor in normal work activities: “health is dearer than money;” “I’ll be healthy – and I’ll get wealthy;” “a sick wife is not nice to her husband.” Harmony was associated with the qualities not only of the world, but also of the peasants themselves, so health was dependent on morality: “A sinful body eats the soul” (Dahl, 1994).

Accordingly, illnesses were perceived as a violation of the existing harmony and order, a consequence of either the peasant’s own misdeeds or those of the peasant community. In the *Domostroi*, a sixteenth-century manual, along with recommendations on household management and child rearing, it is stated that illness is sent by God and that it is wrong to try to cure oneself with medicines; instead, one should pray for forgiveness and lead a good Christian life. The text also mentions folk healers, but their activities are condemned and readers are forbidden to consult them (Kolesov, 1994, p.15).

By the nineteenth century, despite the development of official medicine, peasants far more often used the services of folk healers. In this article, we dwell on the role of the doctor, the herbalist, and the priest as actors in the representation of illness. These three figures are associated with fundamentally different ways of understanding the course of illness and the causes of recovery. The healer, the priest, and the doctor interpreted and, consequently, constructed illness in different ways, and accordingly used different cures relevant to their realities. Following Mol, we assume that reality does not precede practices but is constantly constructed by them; that is, practices simultaneously produce statements about realities and the very realities they describe. Realities are interpreted as causes of these statements: “Realities are not explained by practices and beliefs but are instead produced in them. They are produced, and have a life, in relations” (Law, 2004, p.59). However, let us emphasize that the realities produced by the practices of doctors, priests, and herbalists may well have overlapped with each other.

We begin our description with the work of doctors, whose role in the period under study was the least visible. Following the folk proverb “the soul that goes to doctors does not live,” peasants believed that doctors could not normally help or treat a sick person, since the nature of illness lay beyond their understanding: it was the soul that was sick, not the body. Therefore, peasants turned for help to priests and healers rather than doctors. There were two main reasons for this. First, the number of certified doctors was insufficient to provide medical care to all villagers. The historian N.A. Minenko (1979) has noted that rendering assistance to the population in preserving and restoring health came to the serious attention of the state only in the eighteenth century. Even by the end of the nineteenth century, there were not enough doctors for the peasants. For example, the population of 348,822 from Ishim district (Tiumen province), scattered over an area of 37,604 square *versts*,^1^ was served by just seven district doctors. On average, each medical district had about 50,000 inhabitants and an area of about 5,600 square *versts*. There were only 60 beds for the whole district (including 15 beds in the optical hospital in Petukhovskoe village), meaning there were 5,736 people per bed (Proceedings…, 1913, p.1). Similar information is available for other regions of the Russian Empire (Krutovsky, 1911; Semenova, 2010). The traveler and researcher N. Leskov, traveling throughout Olonets province in 1892-1895, noted that only one doctor was appointed for the huge area (the size of “half a European state or even more”); in some villages there were paramedics, but, as Leskov noted, they were “ignorant” when it came to treatment (Leskov, 1894, p.24). The equipment in hospitals also left much to be desired. Here, for example, is a description of a district hospital:

It is located on the outskirts of the highland part of Tobolsk, in a rented house. The house is wooden, roomy, newly renovated, and two-storied, [with] five rooms upstairs and downstairs, not counting corridors and kitchens. On the lower floor there are: a vestibule (a waiting room), a doctor’s office for receiving outpatients, a pharmacy, two women’s wards, a room for a midwife, a room for a paramedic, and kitchens. On the upper floor there is an operating room, two large male wards, a room for servants, one room turned into a storage space: the kitchen of the second floor has been turned into a bathroom. The wards are ventilated by large window vents. There is no warm water-closet, morgue, or bathhouse. The servants are a cook, a nurse, and an attendant. The doctor is attended by a midwife and two paramedics, one in Tobolsk at the hospital, the second at the paramedic station in the village of Baikalova (Proceedings…, 1913, p.51).

Secondly, doctors did not inspire trust: “There was one paramedic for the whole district, and few people turned to him for help, they did not believe in medicine, they were afraid that it would ‘slaughter’ them” (Goncharenko, n.d.). As a rule, there was a lack of mutual understanding between doctor and peasant, which caused skepticism towards official medicine: “Of course, such paramedics only profane medicine, forcing the population to turn to local folk healers” (Postnikov, 1919, p.54). For example, women in labor believed that medical instruments and examinations were sinful and indecent (Bezgin, 2004). The people’s wariness of pharmacies and doctors was reflected in proverbs, such as the aforementioned adage that “the soul that goes to the doctors does not live” (Dahl, 1994, p.274). Accordingly, the picture of disease constructed by official medicine, as a rule, did not intersect with the reality shared by peasants.

Peasants turned to doctors only after initially trying all available home remedies: “They would give the sick person peppermint, throw him on the stove, cover him with fur coats, and rub in steamed radish. They will also turn to a folk healer, and if this does not help, they will seek advice from a landowner, priest, teacher; only as a last resort, on the urgent advice of one of these persons, they will take the sick person to the hospital” (Popov, 1903, p.100).

Despite fairly widespread knowledge of epidemic diseases, doctors were shocked by the lack of elementary hygiene in villages: “Various household utensils are often washed in the same pond, and at the same time it serves as a source of water for food. The result of poor and often disgusting water supply is the spread of intestinal diseases: dysentery and typhoid” (Popov, 1903, p.17). When bleeding from serious wounds, peasants turned to “grandmothers and medicine women” and rarely went to paramedics for help. As a consequence of such treatment, women often died from blood loss (Makarenko, 1897, p.74-78). While doctors regarded such examples as manifestations of ignorance, we see in this the reflection of a different worldview, where the reality of doctors could not compete with the representation of illness offered by folk healers, priests, and the peasants themselves.

The situation in central Russia began to improve after 1864 as a result of the formation of zemstva (local bodies for rural self-government). Zemstva purchased medicine and distributed it around a network of stations, thereby replacing traveling doctors. Although such stations had only been formed in 46 districts out of 96 by the end of the nineteenth century (Zhbankov, 1894, p.515), they helped to improve healthcare in rural regions. The activities of zemstvo medicine included providing medical help for the rural population, introducing obstetrics, fighting infectious diseases (especially syphilis and smallpox), implementing sanitary supervision and measures, providing sanitation statistics, spreading knowledge about hygiene, and offering material support for zemstvo doctors (Frenkel, 1913, p.110-114, 225-227). However, zemstva were only introduced into some parts of the empire, not touching areas with predominantly non-Orthodox populations or territories in which agriculture was poorly developed (including the north and Siberia, the areas that provide the material used in this article).

In areas lacking zemstva, the main actors constructing the image of illness were priests and folk healers. For peasants, the reality with which these healers and holy men dealt was more significant than the reality in which doctors lived. This situation was not unique to Russia: the authority of folk medicine and religious officiants can be seen in other places, too (Candrea, 1999; O’Connor, Hufford, 2002). The story of pestilence as God’s wrath and punishment was traditionally used by the Russian Orthodox Church for propaganda purposes. Disease was interpreted by priests as a result of some misdeed on the part of the parishioner. For example, during the cholera epidemic of 1848, “St. Nicholas appeared to someone in the woods in the form of an old man and ordered him to leave his colorful and dapper clothing, saying that if this was not done, the devastating disease would last for a long time” (Lukanin, 1869, p.474). While priests viewed illness as a consequence of man’s sinful nature, herbalists considered it to be the result of evil spirits or the negative practices of other people. This semi-pagan belief in evil spirits as a source of disease fit into Christian views. The vitality of folk ideas about the spirits of disease contributed to comparisons between the sick, suffering from a curse, and demon possession, as described in the Gospels, which Christ treated by casting out the demons. As a rule, the herbalists also accompanied their communications with the sick person with Christian symbols – the sign of the cross, prayers, and incessant mention of God and the saints (Dmitrieva, 1999, p.768).

The healer and his actions were endowed with the power to revive hope in the relatives of the patient and mentally transfer the victim from the space of the dead back to the space of the living, thus inducing families to care for and feed them again. The boundary between this world and the world beyond was relative, and the concepts of “viable” and “unviable” could be expanded by folk healers, who were able to see and feel the signs of death and recovery and could persuade the relatives of patients not to lose hope (Ransel, 1996, p.112).

In the minds of peasants, there was therefore a very extensive class of diseases based on curses, the violent transfer of a person to the space of the dead. “A curse, as a consequence of an evil deed by an ‘unkind person’, manifests itself, according to the people, in the form of a health disorder, a change of state of mind for the worse” (Makarenko, 1897, p.84); “cursing was done out of hatred, out of spite at the sick person, at the request of others, for money, or even simply out of love for such art” (Popov, 1903, p.25). Here is further ethnographic evidence: “The basis of cursing is always intentionality, which stems from the personal ill-will of the ‘unkind person’ towards his neighbor, which brings – willingly or unwillingly – his anger. Secondly, out of self-interest, tempted by the reward for what he has done, the ‘unkind person’ decides to cast a curse” (Makarenko, 1897, p.84). Sorcerers could cause a variety of diseases: they could “drive a demon into a person,” “shrivel a person to the bone,” and cause hiccups, languor, paralysis, and sexual impotence. “They are also the culprits of widespread diseases and fevers, cause drunkenness in men, and take milk away from women” (Popov, 1903, p.25). It was believed that only a “knowledgeable person” could cure a curse, such as the sorcerers themselves, who could be forced or begged to remove it.

There were two types of curse: temporary (for a few days), from which one could recover, and permanent (incurable). There were also “love spells.” It was supposed that with the help of a sorcerer it was possible to “bewitch a girl” or “exhaust a boy with longing.” The curse was always cast secretly; it could be transmitted “by the wind” or via a wide array of objects (Makarenko, 1897, p.86). Another very common interpretation of the causes of illness was the evil eye: “Disease comes not only from colds and the like, but from the evil eye … and other things” (Makarenko, 1897, p.59). The evil eye, unlike curses, could be treated by ordinary women.

Thus, curses and the evil eye, as conceived by folk healers and peasants, could act as the framework within which almost all diseases, except for those resulting from the transgressions of specific peasants or the peasant community, were understood. The valued skill of the healer and priest consisted in the possibility of communication with the world of spirits or the sacral world, with the help of which it was possible to recover.

## What was the treatment?

Since illness was seen by both herbalists and priests as a disturbance of harmony and the natural order, efforts to heal had to be directed toward restoring that order. Both herbalists and priests in the nineteenth-century Russian Empire viewed the patient as part of a particular system, the functioning of which had to be restored. Mol describes an important process in medicine: the shift of doctors’ attention from the organ or body part to the patient during surgery, calling this process “ontological politics.” We cannot describe in detail the ontological politics in the period under study because of the limited source base, but we agree with Mol’s thesis that illness does not exist in itself, but is produced by the efforts of peasants, families, communities, herbalists, folk healers, and priests. While Mol has argued that modern medicine not only treats an organ, but also views the patient as part of something larger, the folk healer and priest needed no proof of this. Moreover, the efforts of healers were not so much directed toward the recovery of a particular patient as toward the restoration of a certain harmony, an order.

Diseases were perceived as anthropomorphic and zoomorphic beings: “Disease, in the peasant’s opinion, cannot in any way consist of particles of air. It is the body and spirit of some mythical creature that has the power to be visible and invisible to people’s eyes” (Sherstobitov, 1865, p.56). Swamps, forests, and rivers were considered to be the usual residence of ailments, or rather their spirits. They hovered in the surrounding space, approaching a person at night. Such diseases included the plague and cholera. Plague and cholera, according to popular beliefs, became visible by taking the form of women, especially distinguished by bad clothes and aged, ugly faces (Makarenko, 1897, p.61-62). Some diseases, especially fever, appeared to people in the form of a sparrow, a lamb, or a snake, as M.I. Osokin (1856) wrote of the beliefs of inhabitants in Malmyzhsky district (Vyatka province) in the mid-nineteenth century. The idea of fevers as daughters of the biblical king Herod was widespread. Twelve fever sisters, whose names signified the symptoms of the disease, often appear in representations (Dmitrieva, 1999, p.767). The population of Gorsky parish in Perm province represented fever “in the form of some wench” (Shishonko, 1884, p.156). Fever was also known among peasants as a *kumushka*, a living creature endowed with incomprehensible, supernatural power: “Before the *kumushka* starts to shake someone, they advise this person to get on a humble horse and ride into the field. You must not be afraid. The *kumushka* will rush in pursuit. It will throw itself on the horse like a beast, roar and introduce itself in every way. You must endure, ride in silence – then it will leave” (Makarenko, 1897, p.62).

Protection from spirits required protective measures, such as praying or making the sign of the cross before going to bed (Dmitrieva, 1999, p.766). Researchers have noted that there was a kind of “magical prophylaxis” at play – a desire to keep the spirits of disease out of houses not yet infected during epidemics. Moreover, the main role here was played by the community. Such methods could bring real benefit because they separated the infected from the healthy, introducing a quarantine regime into the village. All these precautions, according to peasants, saved them from disease. For example, during smallpox epidemics, locks were hung on the doorposts of houses currently free of sick people. This meant that there was no entry for the disease. A preventive measure of a public nature was so-called “plowing.” It consisted of drawing a magic line, beyond which the disease should not spread. This line was drawn with a plow, to which young girls or widows, sometimes naked, were harnessed. Thus, at the beginning of an epidemic, either individual houses or the whole village were marked out to prevent the disease from spreading from neighboring villages. If the disease appeared in the village, the sick had to stay at home behind the line, inside the magic circle. When “plowing,” the women and girls would wave pokers and brooms to drive the disease away by scaring the spirits (Makarenko, 1897, p.81-83).

If it was not possible to protect against spirits, it was necessary to remove the spirit from the body of the patient. That is, the anthropomorphic nature of the disease entailed ways to tackle it. First of all, the disease could be killed. That is why in fairy tales not only living but also dead water was used to revive a dead person or cure a disease. At first glance, it seems strange that a sick person should be given dead water. The point is that wounds and diseases were perceived as living beings, so it was necessary to kill them first with the help of dead water and then revitalize the patient with living water. In A.N. Afanasyev’s collection of Russian folk tales, the number of such narratives is quite large, summing 25 in total (Afanasyev, 1985).

According to peasant beliefs, it was possible to negotiate with the spirit of an illness or order it to leave. Therefore, researchers distinguish verbal magic as a special type of healing magic, based on belief in the power of words, on the belief that it is enough to say certain words to cure diseases. In folk practices of banishing disease, these spells were an integral part of healing. Many folk treatment techniques were accompanied by “spell words and whispers,” which were also remnants of pagan prayers. Any phrase, insignificant at first glance, had a certain meaning. There were detailed rules for reciting incantations (Chumakova et al., 1999, p.15). Ethnographers recorded hundreds of such spells. Some of them are published, others are stored in archives. Most of the recorded incantations are for burns, bleeding, bruises, teeth, throat diseases, scabies, fever etc. Especially common were spells for children’s diseases. A spell against the evil eye was used not only for children, but also for adults, if the cause of illness was the evil eye of an envious person (Dmitrieva, 1999, p.767).

Most often, the recitation of incantations was accompanied by certain actions designed to strengthen the magical role of the words. Water was frequently used; milk, tea, and oil, less so. When reciting incantations, a ladle or some other vessel was filled with clean water. Then a cross was drawn on the bottom of the vessel while a spell was spoken. After that the enchanted water was splashed on the face of the sick person or the stricken body area. This procedure was usually done three times. In some cases, the water was thrown out onto crossroads: it was assumed that the disease would be thrown out together with the water (Dmitrieva, 1999, p.767).

Prayers were a variant of spells allowed by the Church. Demons were usually exorcised by village priests, while prayers influenced healing spells. Under their influence, the texts of many ancient incantations were changed, mixing pagan elements with Christian rhetoric:

They took animal fat, melted it into a liquid, and poured it into a cup with cold water in small portions, while reciting a prayer that I memorized: ‘On the ocean sea, on an island in Buyan,^2^ there is a pillar, on that pillar sits a maiden, she is not afraid of anything. And you, commotion, unclean spirit, go to the reeds, to the swamps, to the prickly thorns, to the prickly briar, there to litter, there to root, from this hour, from this day the slave of God (name of the patient) will not know the age of ages, amen’ (Goncharenko, s.d.).

Another way to get rid of a disease was to scare it. The most common magical method was using items with a sharp, unpleasant taste or odor, such as imbibing bitter, disgusting drinks or filling a room with acrid smoke. It was not without reason that peasants believed that the effectiveness of a medicine was determined by the degree of its bitterness. Sometimes they tried to frighten the disease with gunshots and unexpected screams.

In addition to incantations, various objects, associated both by peasants and folk healers with the sacred, were used to communicate with the disease. Relics of saints and miraculous icons were most important in this regard. In connection with the veneration of shrines, the custom of vows (promises made to God) arose. If an illness became chronic, the sick person would make a vow to walk, for example, to the Kiev-Pechersk Lavra, or even to Mount Athos or Jerusalem, in order to worship the holy relics. In the Russian north, it was customary to erect a cross upon making a vow (Dmitrieva, 1999, p.770). In popular opinion, those who received extreme unction and had then recovered were considered dead people who had returned from the other world. In most Russian regions, this sacrament was avoided: those to whom it was given and who then recovered made severe vows, such as abstaining from meat or marital relations (Bernstam, 2000, p.162). The most ordinary things could acquire miraculous power if they were consecrated on great feast days, such as baptismal water and “holy herbs” collected on St. John’s Day or during the Pentecost.

Objects could also be used in the magical transfer of disease to other people, plants, animals, earth, or water. For example, a sick person might be rubbed with a towel or a shirt, which would then be thrown on the road. The disease would pass to anyone who picked up the item. Sometimes, it was enough to take a sick person’s belt to a road, or better still, to a crossroads. There were other ways of transmitting diseases, such as weaving rings out of thread while making an incantation. Once someone had done this, they would cast these rings on the ground at a place where the person on whom they wanted to “put the collar” would pass: it was supposedly enough to step over or touch such a ring to fall ill. Only the folk healer who knew the right spell could help that person recover (Makarenko, 1897, p.74-78). Here is further testimony regarding a similar practice: “If a sick person lies between two trees in the forest and on one of them leaves a belt with a copper ring, and a woman ties a red handkerchief, the disease will be transferred to the one who takes these things, while the one who left them will recover” (Makarenko, 1897, p.74-78).

There were also methods of transferring illness to water. For example, a sick person could take a slice of bread, salt it heavily, and throw it into the water, reciting magical incantations asking for the water to give them good health. A simpler method of recovery with the help of river water was also widely known: in early spring, when the ice melted, girls would rush to the river to bathe in the waters in order to receive good health and beauty (Dmitrieva, 1999, p.766-767).

Disease could also be transmitted to soil. It was believed that “this very element is so holy and pure that it does not contain anything impure and especially hostile to people” (Maximov, 1991, p.325-326). That is why damp earth was applied, for example, to snake and insect bites. Even smallpox was treated with it, using the following procedure: mud from a pigsty would be rubbed on a shirt, which would then be put on the sick person (Makarenko, 1897, p.76-79). During epizootics, a ditch would be made at the edge of the village. It was believed that an animal passing through the ditch would transfer all its diseases to the earth and recover. Folk healers used earth from graves for healing purposes, while sorcerers used it to “inflict” diseases. Earth was often sewn into a purse with incense and worn together with a cross to protect against evil spirits. The same amulets, tied in cloth knots, were hung above the doors of people’s houses to keep evil spirits away.

The same cleansing power was possessed by fire. When someone from a household came down with a fever, a fire would be lit on the floor in the center of the room, through which all the healthy members of the family would cross. In case of an extensive epidemic, public measures would be taken: all residents would gather in the street and build a small fire, through which both the healthy and the sick would pass (Shane, 1989). Often, disease was transmitted to a tree. In the case of prolonged illness, a child could be dragged through a previously made cleft: oaks were used for boys and birches for girls (p.77).

It is necessary to mention separately the wide use of baths for therapeutic and prophylactic purposes. Ethnographers noted that Russians had a passion for baths; they often steamed and washed children, especially at the slightest cry or when suspecting the evil eye. Baths in the eyes of the peasant had unquestionable therapeutic authority. In particular, folk healers often recommended bathing to cure disease, for which they used special incantations. Magical formulas were also used during ordinary washing: “like water off a goose,” they would say, referring to the disease, while dousing a patient with water (Dahl, 1994, p.274). In the fairy tale The Medicine Fox, the fox undertakes to revive an old woman in the bathhouse, washing her bones (Afanasyev, 1985, p.18).

Herbalism was widespread. The study of the practice of using herbs in folk medicine shows that they were valued not so much for their pharmaceutical properties as for their ability to ward off unwanted spirits. For example, a branch of mountain ash provided protection against curses (Dmitrieva, 1999, p.768), while herbs collected on Kupala Night (St. John’s Eve) had special healing powers.

Thus, ailments were imagined by peasants, herbalists, and priests as having anthropomorphic or zoomorphic features. This image was both the cause and the consequence of established healing practices involving killing, entreating, or frightening the ailment. In addition to human actors, healing practices were associated with the involvement of other objects: baths, religious objects, and elements of nature (fire, water, and earth) sacralized by peasants.

## Final considerations

In the nineteenth century, the Russian Empire was a vast state whose various regions had distinct socioeconomic conditions. This article has focused on the Russian Orthodox peasants who lived in the north and in Siberia. By choosing this geographical framework, we have concentrated on peasants who mostly lived outside the medical care system that came into being as a result of the zemstvo reforms of 1864. Peasants in the northern and Siberian provinces maintained traditional worldviews about health, illness, and treatment longer than their countrymen in central Russia.

For us, it was important not to look at concrete forms of treatment, which have received considerable attention from historians of medicine, but to turn our attention to the experience of the patients (peasants) themselves, along with folk healers and priests – actors who wielded authority among the peasant population. Putting the question in this way allowed us to expand the conception of diseases from which the peasantry suffered. It is an approach we believe will be of interest to historians of medicine who research folk beliefs about health and treatment. Using Annemarie Mol’s conclusions, we examined illness as an assemblage, a compilation of the views of various actors: doctors, priests, folk healers, peasants, family members, peasant communities, religious objects, verses, and items used in daily life. Thus understood, illness was conceived as a consequence of either blending the worlds of the living and the dead (damage) or the action of anthropomorphic/zoomorphic entities caused by human sin or the intentions of other people or forces. Consequently, treatment proposed a return to the “natural” (from the peasants’ perspective) order of things, to the world of the living, and the destruction or persuasion of the anthropomorphic/zoomorphic entities concerned. As such, while some practices may have been as effective as modern medicine, they had a different meaning, based on a different conception of disease. We believe that studying not only the practices themselves, but also the meaning that patients put into them, could be useful to researchers of the history of medicine, as it will expand the understanding of what can be considered health and clarify the factors that influence its maintenance.

Untouched by the zemstvo reforms, village doctors in the northern and Siberian regions were the least influential actors in many communities, since they were lacking in numbers and the peasants distrusted their work. A similar situation has been noted by researchers in other regions. Like their counterparts in India, Africa, and distant parts of the European north, the doctors we describe in this article had to maneuver between the medical practices they had studied and the practices that would be accepted by their patients.

Alongside a recent monograph dedicated to the development of medicine in the rural European north between 1800 and 2000, our research shows the “exceptional difficulties of distance and isolation” (Connor, Curtis, 2011, p.84) inherent to practicing medicine in the northern and Siberian regions of Russia. However, while this monograph discusses the creation and development of health systems and how medical workers understood their tasks in these distant northern regions, we have striven to emphasize the role of priests and folk healers in constructing conceptions of illness, as well as the conceptions of the patients themselves.

Our work here calls on those historians of medicine studying communities in which traditional practices coexist with the “civilizing mission” of official medicine to consider actors other than doctors who also had an impact on patients: one might mention here village healers, shamans, and the representatives of state or local religions. Their authority could be greater than that wielded by doctors. In such conditions, a doctor could only act as part of a system, one that he or she might influence but not fundamentally change. The attitudes of patients toward doctors and official medicine in such regions (Siberia, the northern regions of Russia in the nineteenth century, certain parts of Africa, India, and Latin America, remote areas of northern Europe) were a factor that influenced treatment processes. Following Mol, we recommend paying attention not only to patients, but also to members of their families and local societies (peasant communities, in the case of the Russian Empire). We also believe it is important to take into account traditional conceptions of health and illness, along with the place these conceptions occupy in the microcosm of a given community.

## NOTES

1 A *versta* was 1.068km.

2 Buyan is a folkloric island-city said to contain the center of the world.
